# Fear of Terror and Psychological Well-Being: The Moderating Role of Emotional Intelligence

**DOI:** 10.3390/ijerph15112554

**Published:** 2018-11-14

**Authors:** Syed Asad Ali Shah, Tian Yezhuang, Adnan Muhammad Shah, Dilawar Khan Durrani, Syed Jamal Shah

**Affiliations:** School of Management, Harbin Institute of Technology, Harbin 150001, China; tianyezhuang@hit.edu.cn (T.Y.); adnanshah486@gmail.com (A.M.S.); dilawar.khan@live.com (D.K.D.); jimmikakakhail@hotmail.com (S.J.S.)

**Keywords:** fear of terror, psychological well-being, emotional intelligence, adolescents, Pakistan

## Abstract

The purpose of this study was to empirically explore whether or not the level of emotional intelligence of adolescents mitigates the potential adverse effects of the fear of terror on their psychological well-being. Data for this study were collected through a voluntary survey from a sample of 385 adolescents residing in the terrorism-affected provinces of Pakistan: Khyber Pakhtunkhwa (KPK) and Balochistan. The results from the structural equation modeling revealed that fear of terrorism had a significant negative relationship with the psychological well-being of adolescents. The study results further revealed that emotional intelligence significantly moderated the relationship between the fear of terrorism and the psychological well-being of the adolescents. Therefore, the negative relationship was stronger for those with low emotional intelligence and weaker for those with high emotional intelligence. This study also discusses several practical implications along with suggestions for future research.

## 1. Introduction

The impression of terrorism is not limited to a specific aspect of life. Terrorism has a widespread impact on the entire society that disrupts the normal routine of a functional society by spreading fear and terror among its inhabitants particularly in regions that have a history of being vulnerable to terrorist attacks. The Middle East, South Asia, and South East Asia are a few such regions that have been the target of frequent terrorist attacks [1,2]. Terrorism is usually defined as the use of violence or threats intended to generate a general climate of fear among the population, which aims to carry out a specific political objective [3]. Terrorists frequently target places that are considered easy targets such as educational institutions, clubs, transportation, and shopping malls since these places have comparatively little formal security and high levels of population concentration [4]. Terrorist attacks on soft targets especially education institutes have been very frequent in Pakistan with some of them resulting in heavy casualties such as the devastating attack on the army public school in Peshawar on 16 December 2014, which killed 145 innocent schoolchildren along with many teachers [5]. This is one of the numerous horrific terrorist attacks on educational institutions that were carried out in Pakistan during the past two decades or so (for details, see the Human Rights Report [6]).

Adolescence is the stage of life that is characterized by rapid social, physical, and psychological changes. These changes are likely more rapid in adolescence when compared to other periods of life [7]. War, conflict, and acts of terrorism are a few of the most painful and shocking events that may not only influence the daily well-being of these adolescents but also undermine their psychological functioning while they are already going through various social, physical, and psychological changes. Such events of terrorism and conflict produce a sense of insecurity among societies that eventually becomes the cause of anxiety, depression, fear, and mental problems [8,9,10,11]. These negative consequences may have a widespread impact on various aspects of life including the deteriorating academic performance of those adolescents whose schools had been targeted in the past or face potential threats from various terrorist groups [12]. Proclaimed threats of possible terror attacks on educational institutions have become a common phenomenon in Pakistan since these terrorist organizations assume the existence of educated youth to be a threat to their own existence [6]. Traumatic events like terrorism are not limited to specific regions but are becoming the cause of growing public health problems all over the world. Turning towards the context under consideration in this study, we are of the view that such a threatening environment has the potential to adversely affect the psychological well-being of adolescents residing in the terrorism-affected areas of the KPK and Balochistan provinces of Pakistan.

The existing studies of terrorism in the context of adolescents have mostly focused on the USA after 9/11 and very little is known about how the youth of developing countries respond to acts of terrorism [13]. Pakistan is a country where frequent terrorist incidents have created a catastrophic atmosphere especially those adolescents who are attending schools and colleges. The teachers, parents, and health care providers continuously struggle to help adolescents cope with such heightened terrorism, which generated stress and fear. Considering the importance of finding ways to help adolescents cope with the terrorism-induced strain, it is essential for researchers and the general public alike to focus on understanding the impacts of terrorism on adolescents and children [14]. More specifically, it is becoming increasingly important to explore the effects of terrorism-induced fear on the adolescents’ psychological well-being and identify the means that can help mitigate the negative effects of the fear of terrorism in the given context, which has not been explored in previous literature.

Parallel to the above arguments and taking the conservation of resources (COR) theory as a theoretical background, this study aimed to fill the gap in knowledge by linking the fear of terror to adolescents’ psychological well-being. Moreover, the previously ignored but intuitively intriguing moderating role of emotional intelligence on the relationship between fear of terror and psychological well-being was examined.

## 2. Literature Review

### 2.1. Fear of Terror and Psychological Well-Being

The psychological well-being of adolescents refers to being happy and satisfied with one’s life, the absence of negative emotions, and the presence of positive emotions. Furthermore, psychological well-being also indicates the existence of very good academic success, physical health, social support, skills, and purpose in one’s life [15]. Research on well-being has largely focused on adult populations in most developed economies [16,17]. The self-reported subjective well-being of adolescents and school-children in developing countries like Pakistan is underexplored specifically in relation to terrorism. Very few studies have examined these relationships in other contexts [18]. Previously, the research on terrorism has shown that exposure to terrorism strongly affects the positive psychological state of adolescents [19,20,21]. Therefore, we believe that adolescents’ terrorism-related fear may affect their psychological well-being while going to school or by observing such incidents. The fear of terrorism has been found to induce many undesired psychological states in individuals such as increasing their level of anxiety [22], depression [23], insomnia [24], and other mental health [25] problems. Moreover, these horrendous events of terrorism may have an extremely harmful impact on their ability to manage their normal life developmental tasks, which, in turn, affects their psychological well-being [26,27]. The claim that terrorism and the fear that stems from it have a strong impact on adolescent psychological well-being are also in line with previous studies by Ghasemi [28] and Yahav [25].

To further establish the relationship between the fear of terror and adolescent psychological well-being, we based our arguments on the COR theory [29,30]. The theory posits that individuals strive to obtain, retain, and guard their valued resources. Resources in the current context can be intangible entities that have their own intrinsic significance for an individual (for example, intangible psychological resources such as health, close attachments, inner peace, and self-esteem) [31]. The fear of terror translates into the worries of individuals for losing their valuable resources (i.e., life and health of friends, family members, and themselves), which, in turn, provokes stress and may consequently lead towards anxiety, depression, and post-traumatic stress disorder [22,23,24,32]. This fear may also lead individuals into a state of physical and psychological resource loss or strain, which lowers their well-being [30,33]. Other studies have also led to similar findings that a loss of resources is positively related to psychological strain in adolescents [34,35]. Similarly, Reade and Lee [36] found that individual exposure to ethnopolitical conflict resulted in psychological resource loss. Hence, we proposed the following hypothesis.

**Hypothesis 1** **(H1).** *Fear of terror is negatively related to psychological well-being.*

### 2.2. Moderating Role of Emotional Intelligence between Fear of Terror and Psychological Well-Being

This study proposes that emotional intelligence (EI) in adolescents can be considered as one of the abilities that can help mitigate the negative effects of terrorism fear on their psychological well-being. EI has been defined as “the set of abilities (verbal and nonverbal) that enable a person to generate, recognize, express, understand, and evaluate their own, and others, emotions in order to guide thinking and action that successfully cope with environmental demands and pressures” [37] (p. 72). The construct of EI consists of four inter-related aspects of emotion including perception, understanding, facilitation, and regulation of emotions [38]. It represents the individual differences of ability and capacity to observe and recognize emotions (in self and others) and use such information for the regulation of their emotions and actions [38,39,40]. Based on the assumption of the regulation of emotions, researchers argue that, by using certain emotional strategies, an individual could reduce negative emotional states while contributing to a positive emotional state [41] when facing an uncertain negative situation such as terrorism and its fear. Therefore, we assumed that EI could not only reduce the negative emotions felt because of the fear of terrorism and its related stress but also aid in the maintenance of positive emotions and psychological well-being despite the negative circumstances [41,42]. The concepts of EI, psychological well-being, and health have attracted the attention of numerous studies in recent years [43,44,45,46,47,48]. Lau and Wu [49] stated that EI is a vital component of ‘social maturity’ that enhances the psychological health of adolescents. Furthermore, EI has been considered as a key factor in mental health and positive well-being [50].

Moreover, drawing from the conservation of resource protection mechanism by Hobfoll [29], we suggest that individuals with higher levels of psychological resources (i.e., emotional intelligence) experience little psychological strain and cope easily with the stress-induced fear of terror by utilizing their extra resource reserves. EI is expected to alleviate the negative effects of the fear of terror and increase adolescent psychological well-being through facilitation, recognition, and management of emotional reactions to the fear of terror and related stress [51]. We further propose that adolescents with a high level of EI would have higher abilities to cope with the resource loss caused by the fear of terror and its related stress since they are more likely to evaluate emotional information and monitor their own and others’ emotions. Hence, it would be conceivable to say that individuals may differently evaluate the fear and threat of terrorism according to their emotional capacity even when facing similar dreadful situations. More specifically, individuals with higher levels of EI would have a better capacity to analyze and regulate the complex emotions of anger and frustration (resulting from the fear of terrorism) by identifying the causes of such feelings [52,53] and, hence, may be less affected by the fear of terrorism. Moreover, studies have also shown that physical and psychological recovery following disasters depends on the abilities of individuals to counterbalance their resource loss [54]. Such findings are also in line with our propositions. Likewise, another study by Urquijo et al. [55] showed the positive relationship between EI and psychological well-being. Therefore, we proposed the following hypothesis.

**Hypothesis 2** **(H2).** *Emotional intelligence moderates the relationship between the fear of terror and psychological well-being.*

## 3. Materials and Methods 

### 3.1. Participants

The data for this study were collected from different schools operating in the two most terrorism-affected provinces of Baluchistan and KPK in Pakistan. The study was cross-sectional in nature with data collected from December 2017 to February 2018. A total of 600 questionnaires were distributed among middle and high school students of the two provinces. Out of the total potential participants, 74% decided to take part in the survey with the consent of their parents and a total of 444 questionnaires were returned. Out of the returned questionnaires, 59 were discarded from the study because of incomplete and missing information and 385 valid responses were considered for the final analysis in which 58% (*n* = 223) were boys and the remaining 42% (*n* = 162) were girls ranging from 12 to 18 years of age. The average participant age was 14.81 years. Table 1 contains the demographic profile of the study participants.

### 3.2. Procedure

In line with the Helsinki Declaration of 1964 and its later amendments to the best of our knowledge, all of the research procedures were performed within ethical standards. Formal approval was obtained from the competent authorities of the schools that participated in the study. A formal consent form was also signed by both the students and their parents after explaining the purpose of the study and ensuring the complete anonymity and confidentiality of the respondents. Before starting the survey, research assistants clearly explained the purpose of the survey and guided them through the procedure regarding correctly completing the questionnaires. Participants were also informed that their participation in the study was voluntary and they could decide to withdraw from the survey at any stage. After the briefing, the research assistants distributed the questionnaires during the scheduled school hours with the permission of the competent authorities. At the end of the survey, the research assistants presented small gifts to all study participants in appreciation of their contribution to the study.

### 3.3. Measures

For the measurement of our study constructs, we used previously well-established and reliable scales. The scale items were slightly modified according to the context of the study. All three constructs were measured on a five-point Likert-type scale ranging from 1 = Strongly disagree to 5 = Strongly agree.

Fear of terror (FOT) was measured by using the 13-item Terrorism Catastrophizing Scale developed by Sinclair and Locicero [56] to assess the ongoing fear of future terrorist attacks. Psychological well-being (PWB) of adolescents was measured through the World Health Organization five-item scale for well-being, which was adapted from the study of De et al. [57]. Lastly, Emotional Intelligence (EI) was measured by using the 16-item scale developed by Wong and Law [40]. Cronbach’s alpha reliability values for the scales used are given in Table 2 on the diagonals in bold.

We also considered two potentially relevant control variables, i.e., age and gender of the adolescents. First, age was measured in years since older adolescents with more experience and greater EI may depict comparatively better abilities to cope with the challenges faced by them in different stressful situations. Second, we controlled for gender since previous research showed that girls became more fearful after terrorist incidents when compared to boys [58].

### 3.4. Analysis

The analyses were performed by using IBM SPSS (version 23.00, SPSS Inc., Chicago, IL, USA) and AMOS (version 23.00, SPSS Inc., Chicago, IL, USA). Initially, exploratory factor analysis (EFA) was performed to check whether or not the items of the scales loaded on their respective factors. After that, confirmatory factor analysis (CFA) was carried out to check the measurement model fitness. Lastly, we applied structural equation modeling (SEM) by using AMOS to test whether or not the proposed relationships were statistically significant. Moreover, we also tested the scales used in the study for reliability, discriminant, and convergent validity.

## 4. Results

### 4.1. Descriptive Analysis

The results from the descriptive analysis including means, standard deviations, and the correlations are shown in Table 2. The results showed that FOT had a significant negative correlation with PWB as expected. Furthermore, EI had a significant positive relationship with PWB. Gender and PWB were also found to be significantly correlated while the rest of the correlations were insignificant.

### 4.2. Model Fitness, Validity, and Reliability of the Study Constructs 

CFA was carried out to check whether the measurement model fit the data well. Several fit indices were also calculated to see if the structural model fit the sample data. All values obtained from the model fit indices depicted that the model fit the data well. More specifically, χ^2^ = 772.176, df = 524, χ^2^/df = 1.474, goodness of fit index (GFI) = 0.892, adjusted the goodness of fit index (AGFI) = 0.877, Tucker–Lewis index (TLI) = 0.970, normed fit index (NFI) = 0.919, comparative fit index (CFI) = 0.972, and the root mean square error of approximation (RMSEA) was noted as 0.035, which all indicated a good model fit [59,60]. Moreover, the CFA estimation showed that the proposed three-factor model was the best fit for the current sample data. All items loaded well on their respective factors with a factor loading greater than 0.5. Table 3 exhibits the values of average variance extracted (AVE), composite reliabilities (CR), maximum shared variance (MSV), and the square roots of the AVEs of all the study constructs. The CR values were greater than the prescribed threshold of 0.7, which indicated a high level of reliability. Values of AVE were noted to be greater than 0.5 for all of our study constructs, which indicated a high level of convergent validity while the square root of AVEs was greater than the inter-construct correlations for all variables used in the study, which demonstrates the high level of discriminant validity [61,62]. Discriminant validity was further established when the values of MSVs were less than the values of AVEs. The results described that all of the MSVs values were less than AVEs, which provides further support for the discriminant validity.

### 4.3. Structural Equation Model

**Figure 1:**
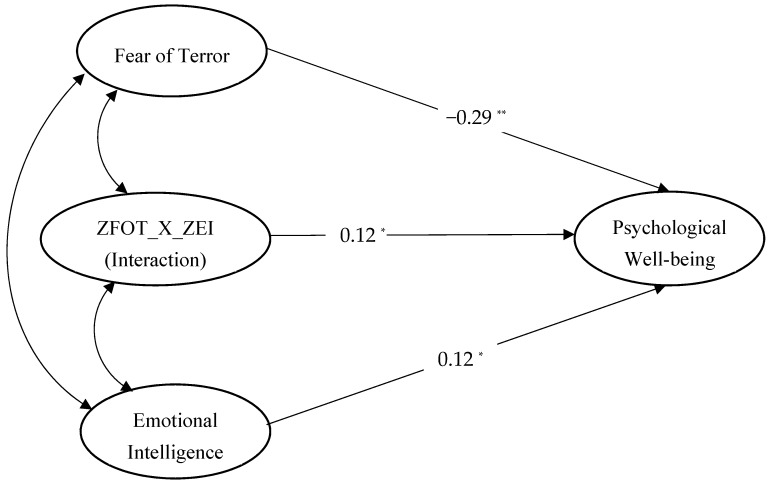
Standardized path coefficients of the structural equation model. Note: ZFOT_X_ZEI = Interaction of the fear of terror and emotional intelligence calculated by multiplying the standardized values of both variables. * *p* < 0.05. **p < 0.01.

SEM techniques were used to test the proposed hypothesis. The maximum likelihood was selected for the assumption of multivariate normality, which researchers consider to be more robust for its non-compliance [63]. According to the SEM approach, we included all of our study’s constructs—FOT, PWB, and EI—in the same model to check their weights and level of significance for the possible relationship among them by using the indications from Schreiber et al. [64]. The structural equation model revealed that it fit the data very well (χ^2^ = 284.847, df = 198, χ^2^/df = 1.439, GFI = 0.935, AGFI = 0.917, TLI = 0.981, NFI = 0.949, CFI = 0.984, and RMSEA = 0.034). The significance level of each path coefficient of the structural model is shown in Figure 1. The standardized estimates presented in Figure 1 show that the path coefficient from FOT to PWB was negative and significant (β = −0.29, *p* < 0.01). Thus, these results support our study’s proposed Hypothesis 1 that FOT has a negative impact on the PWB of adolescents. Furthermore, the results also indicated that the path coefficient connecting EI with PWB was positive and significant (β = 0.12, *p* < 0.05), which illustrates that EI could play a vital role in enhancing the adolescent’s PWB. However, it was seen that the effect size depicted by squared multiple correlations (r^2^) was relatively small (r^2^ = 0.16).

### 4.4. Moderator Analysis

**Figure 2:**
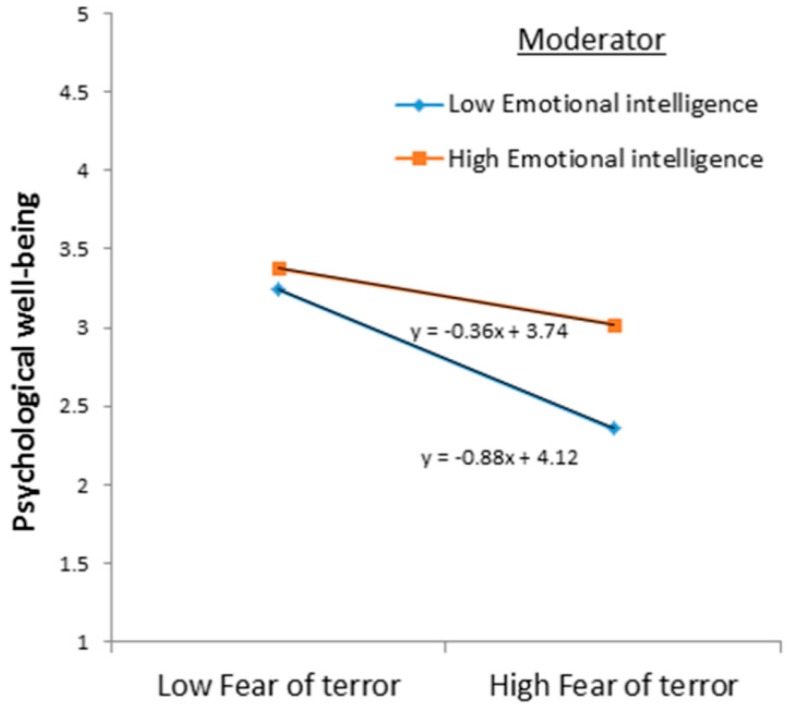
Graphical representation of the moderator emotional intelligence on the association between the fear of terror and psychological well-being.

The moderating effect of EI on the relationship between FOT and PWB was analyzed by calculating the interaction term between EI and FOT and checking the significance of its effect on PWB. The interaction term was calculated by standardizing the imputed scores of FOT and EI and then multiplying it. The results depicted in Figure 1 showed that the path coefficient connecting the interaction term (ZFOT_X_ZEI) with PWB was statistically significant (β = 0.12, *p* < 0.05). Hence, EI significantly moderates the relationship between FOT and PWB, which supports Hypothesis 2. The moderating effect of EI can be further explained through Figure 2. 

It is clear from the graphical representation that the negative relationship between FOT and PWB was weak for those adolescents with high EI and comparatively strong for those with low EI. Thus, this provides support that EI can help mitigate the adverse effects of FOT on PWB.

## 5. Discussion

The objective of this study was twofold: to analyze the possible impact of the FOT on the PWB of adolescents and to explore the moderating role of EI on the relationship between the FOT and PWB. The results showed the existence of a negative relationship between the FOT and PWB. Furthermore, EI was positively related to the PWB of the adolescents and negatively to the FOT. These results of our study are consistent with previous studies related to adolescents [28,30,41,65,66,67]. Moreover, the findings of the study are also in line with COR theory by showing that the fear of terrorism diminished the psychological resources of adolescents, which resulted in a psychological strain and lowered their well-being [30]. Furthermore, consistent with COR theory, the results indicated that EI could act as an emotional resource and thus help the adolescents cope with the strain created by the fear of terrorism and mitigate its negative effects on their PWB. Thus, adolescents with a high level of EI could handle fear-related stress due to terrorism and the resultant challenging emotional situations more effectively by being able to understand and regulate their emotions.

This study contains useful information for educational institutions operating in terrorism-threatened environments as the adolescents studying in those institutions face the continuously existing threat of terrorism, which results in an ongoing fear that needs to be dealt with on a daily basis. Even though the fear of terrorism seems to affect everyone, some of the adolescents may also be reluctant to admit their fear. As a result, they may resort to more passive strategies of coping such as denial and avoidance, disengagement from studies, and reduced interest in daily life activities. The outcomes of the current study are likely to assist teachers, parents, and adolescents. For instance, they would appraise the fear as a normal phenomenon. Therefore, instead of denying and escaping, they would intelligently cope with it by enhancing their level of EI, which would further improve their PWB. To the best of our knowledge, there is no available study that has tried to empirically analyze the association between the FOT and the PWB of adolescents in Pakistan. Moreover, none of the previous studies have examined the moderating effect of EI on the relationship between FOT and PWB. This study attempted to address the identified gaps in the existing body of theoretical and practical knowledge by exploring the previously unexplored relationships in the context under consideration.

The findings from this study have important implications for teachers, students, counselors, parents, and competent authorities alike. However, it might not be possible to completely eliminate the negative effects of the FOT on adolescents’ PWB. These adverse effects, though, can be substantially reduced by making efforts to enhance the EI of adolescents. Studies have shown that EI is not something inherited or inborn. Rather it is a learned skill that can be enhanced through the provision of training [46,67,68]. Therefore, adolescents can be trained in their abilities to cope with such fears intelligently and, thus, overcome the negative effects of terrorism on their psychological well-being. The role of teachers in this regard is of great importance because they have the responsibility to act as mentors for their students. Research in this vein has shown that mentors can assist others in overcoming their unnecessary fears and doubts by providing them with practical insight into the current circumstances being faced [69,70]. The findings of this study suggest that teachers, parents, and counselors should all work toward enhancing the emotional skills of adolescents to manage and reduce their inflated fear of terrorism. To do so, these teachers and counselors first need to understand and develop the skills of EI within themselves so that they can later help to enhance the same skills in their students. It is recommended that specialized training programs may be developed for teachers to first improve their own emotional skills for coping with the stress induced by the fear of terror. Such training would enable the teachers to transfer these skills to their students through daily interaction via formal and informal means. Apart from that, formal training programs and extra-curricular activities should also be designed and particularly tailored to the needs of adolescents. Such an intervention would not only improve their emotional skills but also enhance their positive thinking by keeping them engaged in positive learning activities. These learning strategies should be designed explicitly for particular age groups according to their interests and should incorporate fun, pleasure, and learning at the same time. Activities like this would not only boost the EI of the adolescents but also take their minds off the negative emotion of fear, which improves their PWB. The EI of adolescents could also be enhanced through mindfulness. Studies in this regard indicate that the more significant characteristics of mindfulness are associated with high EI [71,72]. Consequently, mindfulness could be one of the better platforms to develop EI in individuals [72]. Moreover, improved relationships between friends, family members, and parental warmth would also be better able to enhance adolescents EI [73]. It is also equally important for the management of those institutions working in threatened areas to ensure the protection of adolescents studying in their institutions since it would enhance their sense of security and lower their fear of becoming a victim of potential terrorist incidents.

This research has some limitations. The data were collected only through self-reported measures. Therefore, it may contain some social desirability bias [74]. However, we tried to minimize the possibility of such bias by informing the participants about the anonymity of their responses. As mentioned earlier, the samples for the study were selected from adolescents’ residing in the two most terrorist affected provinces of KPK and Balochistan. Therefore, the generalizability of these findings is limited to only those areas that are highly vulnerable to terrorist attacks. For example, these findings cannot be generalized to the broader population or to adolescents who live in areas that are less exposed to terrorism. However, the selection of such a sample was considered necessary since the effects of recent terrorist events on the adolescents of the two most vulnerable provinces of Pakistan have not been investigated widely. Future studies may focus on collecting data from areas that have been less affected by terrorism in the past and compare any differences that may exist in the perception of adolescents that are comparatively more exposed to past terrorism and remain vulnerable to future threats. Lastly, due to the cross-sectional nature of this study, causality could not be determined. Therefore, future research may undertake longitudinal studies with multi-level measurements of the constructs.

## 6. Conclusions

The current research has extended the body of knowledge by providing novel and valuable insights into the psychological experiences of adolescents residing in terrorism-threatened areas. The results of the study showed that the repeated direct and indirect exposure to terrorism incidents can create a fear of terror in adolescents that have devastating consequences by negatively affecting their psychological well-being. In addition, EI can be a valuable skill that can help counter and mitigate the adverse effects of the fear of terror on an adolescent’s psychological well-being. Thus, this research emphasizes the provision of appropriate assistance and training for enhancing EI in adolescents and their mentors. Therefore, despite not having any control over the terrorist incidents, they can still minimize its negative effects. These findings could serve as a foundation for future research investigations that would expand our knowledge regarding the negative psychological effects of terrorism on adolescents, which may eventually help scholars find better ways to reduce and alleviate such effects.

## Figures and Tables

**Table 1:** Demographic profile.

Variables	Dimensions	Frequency	Percentage
Age	12 Years	64	16.60%
	13 Years	23	06.00%
	14 Years	80	20.80%
	15 Years	102	26.50%
	16 Years	29	07.50%
	17 Years	44	11.40%
	18 Years	43	11.20%
Grade	6th	60	15.60%
	7th	29	07.50%
	8th	82	21.30%
	9th	98	25.50%
	10th	33	08.60%
	11th	43	11.20%
	12th	40	10.40%
Gender	Boys	223	58.00%
	Girls	162	42.00%

**Table 2:** Descriptive analysis including means, standard deviations, and correlation between the study variables.

Variables	Mean	SD	1	2	3	4	5
Age	14.81	1.86	-				
Gender	0.42	0.49	−0.002	-			
Fear of terror	3.50	1.23	0.071	0.053	**0.962**		
Psychological well-being	2.82	1.14	0.020	−0.216 **	−0.30 **	**0.891**	
Emotional intelligence	2.83	0.74	0.008	−0.083	0.048	0.132 **	**0.945**

Note. SD = Standard deviations. ** *p* < 0.01.

**Table 3:** Values of composite reliability, maximum shared variance, average variance extraction, and its square roots.

Variables	CR	AVE	MSV	Square Roots of AVE
1. Fear of terror	0.962	0.661	0.083	0.813
2. Psychological well-being	0.892	0.624	0.083	0.790
3. Emotional intelligence	0.945	0.521	0.015	0.722

Note. CR = Composite reliability, AVE = Average variance extraction, MSV = Maximum shared variance.
